# Gender change and stigmatization in late-treated Indonesian children, adolescent, and adult patients with DSD

**DOI:** 10.1186/1687-9856-2015-S1-O56

**Published:** 2015-04-28

**Authors:** Annastasia Ediati, Achmad Zulfa Juniarto, Erwin Birnie, Jolanda Okkerse, Anne de la Croix, Amy Wisniewski, Stenvert Drop, Sultana MH Faradz, Arianne Dessens

**Affiliations:** 1 Diponegoro University, Faculty of Psychology, Semarang, Indonesia; 2 Diponegoro University, Faculty of Medicine, Center for Biomedical Research (CEBIOR), Semarang, Indonesia; 3 Erasmus University Rotterdam – Institute of Health Policy and Management, Rotterdam, the Netherlands; 4 Sophia Children’s Hospital - ErasmusMC Rotterdam, Rotterdam, the Netherlands; 5 University of Oklahoma Health Sciences Center, Oklahoma City, Oklahoma, United States

## 

In Indonesia clinical management of Disorders of Sex Development (DSD) is challenged by limited knowledge and limited diagnostic and treatment facilities. Prior to this study, most patients remained untreated and grew up with ambiguous bodies and doubts about their gender. We investigated patients’ experiences of being raised in ambiguity.

118 Indonesian patients, ages 6 – 41, with 46XX DSD (n=27), 46XY DSD (n=77) and chromosomal DSD (n=14) were compared to 118 control subjects matched for gender, age, and living area. Questionnaires for gender identity, gender role behavior and social stigmatization were translated or designed. The psychometric properties were satisfactory. For patient and control group comparisons, Mann-Whitney U and Fisher’s Exact tests were applied.

The results showed that 7% of the children, 8% of the adolescents and 44% of the adults changed gender, particularly non-diagnosed and non-treated patients with 46XY DSD (81%). 95% of the patients changed gender from female to male, including untreated patients with 46,XX CAH-SV. Compared to control groups, cross-gender role behavior was seen in young girls with 46XX CAH-SV (p=.047) and adolescent girls with different types of DSD (p=.01). In girls with DSD, confusion with gender identity was seen (young girls p=.004; adolescent girls p=.01). Adult men reported past cross-gender role behavior (p=.01) and past problems in gender identification (p=.01) prior to female-to-male gender change.

Children with genital ambiguity (p<.006) and cross gender behavior (p<0.001) and adults with ambiguous bodies (p=.001) and adults who changed gender (p<0.03) suffered stigmatization. Rejection or isolation elicited depression and withdrawal from social activities in girls (p=.002), women (p=.009) and youngsters who had changed gender (p=.02).

We conclude that a high percentage of our patients changed gender. The wish for gender change was particularly seen in patients with progressive masculinization. Patients with DSD who had visible ambiguity in physical and behavioral appearance suffered stigmatization. Teasing and rejection led to strong emotional reactions. Early clinical evaluation and treatment, patient and parent education, and teaching coping strategies will improve quality of life.

